# Relationship between silent atrial fibrillation and the maximum heart rate in the 24-hour Holter: cross-sectional study

**DOI:** 10.1590/1516-3180-2014-1326732

**Published:** 2014-09-02

**Authors:** Marcelo Lapa Kruse, José Cláudio Lupi Kruse, Tiago Luiz Luz Leiria, Leonardo Martins Pires, Caroline Saltz Gensas, Daniel Garcia Gomes, Douglas Boris, Augusto Mantovani, Gustavo Glotz de Lima

**Affiliations:** I MD, MSc. Medical Electrophysiologist, Electrophysiology Service, Instituto de Cardiologia - Fundação Universitária de Cardiologia (IC-FUC), Porto Alegre, Rio Grande do Sul, Brazil; II MD. Cardiologist, Instituto de Cardiologia - Fundação Universitária de Cardiologia (IC-FUC), Porto Alegre, Rio Grande do Sul, Brazil; III MD, MSc, PhD. Medical Electrophysiologist, Electrophysiology Service, Instituto de Cardiologia - Fundação Universitária de Cardiologia (IC-FUC), Porto Alegre, Rio Grande do Sul, Brazil; IV Medical Student, Universidade Federal de Ciências da Saúde de Porto Alegre (UFCSPA), and Scientific Initiation Student, Instituto de Cardiologia - Fundação Universitária de Cardiologia (IC-FUC), Porto Alegre, Rio Grande do Sul, Brazil; V MD. Resident, Electrophysiology Service, Instituto de Cardiologia - Fundação Universitária de Cardiologia (IC-FUC), Porto Alegre, Rio Grande do Sul, Brazil; VI MD, PhD. Medical Electrophysiologist, Electrophysiology Service, Instituto de Cardiologia - Fundação Universitária de Cardiologia (IC-FUC), Porto Alegre, Rio Grande do Sul, Brazil

**Keywords:** Atrial fibrillation, Signs and symptoms, Electrocardiography, ambulatory, Arrhythmias, cardiac, Electrophysiology, Fibrilação atrial, Sinais e sintomas, Eletrocardiografia ambulatorial, Arritmias cardíacas, Eletrofisiologia

## Abstract

**CONTEXT AND OBJECTIVE:**

Occurrences of asymptomatic atrial fibrillation (AF) are common. It is important to identify AF because it increases morbidity and mortality. 24-hour Holter has been used to detect paroxysmal AF (PAF). The objective of this study was to investigate the relationship between occurrence of PAF in 24-hour Holter and the symptoms of the population studied.

**DESIGN AND SETTING:**

Cross-sectional study conducted at a cardiology hospital.

**METHODS:**

11,321 consecutive 24-hour Holter tests performed at a referral service were analyzed. Patients with pacemakers or with AF throughout the recording were excluded.

**RESULTS:**

There were 75 tests (0.67%) with PAF. The mean age was 67 ± 13 years and 45% were female. The heart rate (HR) over the 24 hours was a minimum of 45 ± 8 bpm, mean of 74 ± 17 bpm and maximum of 151 ± 32 bpm. Among the tests showing PAF, only 26% had symptoms. The only factor tested that showed a correlation with symptomatic AF was maximum HR (165 ± 34 versus 147 ± 30 bpm) (P = 0.03). Use of beta blockers had a protective effect against occurrence of PAF symptoms (odds ratio: 0.24, P = 0.031).

**CONCLUSIONS:**

PAF is a rare event in 24-hour Holter. The maximum HR during the 24 hours was the only factor correlated with symptomatic AF, and use of beta blockers had a protective effect against AF symptom occurrence.

## Abstract (pt)

**CONTEXTO E OBJETIVO:**

A ocorrência de fibrilação atrial (FA) assintomática é comum. A identificação da FA é importante e está associada com maior morbimortalidade. O Holter de 24 horas vem sendo utilizado para a detecção de FA paroxística (FAP). O objetivo desse estudo é investigar a relação entre a ocorrência de FAP no Holter de 24 horas e os sintomas na população estudada.

**TIPO DE ESTUDO E LOCAL:**

Estudo transversal realizado em um hospital de cardiologia.

**MÉTODOS:**

Análise de 11.321 exames consecutivos de Holter de 24 horas realizados em serviço de referência. Foram excluídos pacientes com marcapasso e aqueles com FA durante toda a gravação.

**RESULTADOS:**

Foram identificados 75 (0,67%) exames apresentando FAP. A idade média foi de 67 ± 13 anos e 45% eram do sexo feminino. A frequência cardíaca (FC) encontrada nas 24 horas foi mínima de 45 ± 8 bpm, média de 74 ± 17 bpm e máxima de 151 ± 32 bpm. Entre os exames apresentando FAP, apenas 26% apresentaram sintomas. O único fator testado que evidenciou correlação com FA sintomática foi a FC máxima (165 ± 34 *versus* 147 ± 30 bpm) (P = 0,03). O uso de betabloqueadores teve efeito protetor para ocorrência de sintomas na FA (*odds ratio:* 0.24, P = 0.031).

**CONCLUSÕES:**

A FAP é um evento raro em Holter de 24 horas. A FC máxima nas 24 horas foi o único fator relacionado com FA sintomática e o uso de betabloqueadores teve efeito protetor para ocorrência de sintomas na FA.

## INTRODUCTION

Atrial fibrillation (AF) is the most common sustained arrhythmia in medical practice.1 It is often associated with a significant increase in morbidity and mortality, particularly in elderly patients. AF is the leading cause of embolic episodes. Cerebrovascular causes account for 75% of these embolic phenomena.^2^^,^ 3

The diagnosis of AF can be based on the patient's symptoms, but in several cases it may also be asymptomatic. It is sometimes identified by chance or when the patient has a thromboembolic event. Early diagnosis is important in preventing morbidity and mortality, which are largely due to stroke.^4^^,^ 5

Several tools for assessment of AF have been developed, such as transtelephonic monitoring, 24-hour Holter and implantable loop monitoring of electrocardiographic changes.^6^ All these techniques have been evaluated in order to better define the presence of paroxysmal arrhythmias such as paroxysmal atrial fibrillation (PAF). This can be also done within the post-treatment scenario, such as the post-AF ablation setting.^7^^,^ 8 24-hour Holter is useful for correlating arrhythmic events that may be detected with the patient's symptoms.

## OBJECTIVES

The objective of this study was to investigate the relationship between the presence of PAF in 24-hour Holter and the symptoms of the population studied.

## METHODS

A cross-sectional study was conducted on patients undergoing 24-hour Holter monitoring. Consecutive patients from private practice (n = 7974) or from the Arrhythmia Ambulatory at Instituto de Cardiologia (n = 3347), from 1998 to 2007, for whom the test was solicited, were included in this analysis.

Two examiners with experience in assessing arrhythmia reviewed and reported on all the tests in order to identify the presence of PAF. Patients who presented PAF episodes that lasted for more than 30 seconds were included. Patients were instructed to carefully fill out their diaries, which were reviewed regarding the duration of symptoms. Palpitations, dyspnea, dizziness and chest pain reported by the patients were considered to be AF symptoms. The symptomatic group was formed by patients who experienced at least one episode, associated with symptoms that occurred together with the PAF episode.

The software used for analysis on the Holter tracings was DMI (Diagnostic Medical Instruments; Holter Eclipse Analyzer, AR-200 software ALT V5.08B, Burdick Inc, United States) and Cardio Sistemas (Cardio Sistemas Comercial e Industrial Ltda, São Paulo, Brazil).

An analysis on the factors associated with increased symptomatic sensitivity to arrhythmia and the period of the day in which the events occurred was performed. The data were collected from the ambulatory Holter system. Patients with both a permanent pacemaker and permanent AF throughout the recording were excluded, as well as those that presented PAF lasting for less than 30 seconds. The maximum, minimum and mean heart rates used were those obtained in the 24-hour analysis. All of them were duly revised. The examiners analyzed and confirmed the pauses that were greater than two seconds. 

The database was stored in Microsoft Excel 2000. The significance level used was P < 0.05 with a statistical power of 80%. Continuous data, such as episode duration and frequency of episodes, were analyzed by means of Student's t test. Analysis on the differences between categorical variables, such as the symptoms during PAF events, was performed using the chi-square test (Fisher). For asymmetrical variables, the Mann-Whitney test and analysis of covariance (ANCOVA) for repeated measurements were used. Logistic regression was performed to identify factors that increased the odds of symptomatic PAF. The statistical analysis was performed using the Statistical Package for the Social Sciences (SPSS) v.12. 

This study was approved by the Research Ethics Committee of the Institute of Cardiology of Rio Grande do Sul, University Cardiology Foundation (Instituto de Cardiologia/Fundação Universitária de Cardiologia, IC/FUC). 

## RESULTS

11,321 Holter tests were performed and analyzed. PAF was found in 75 cases (one test per patient), which represented a prevalence of 0.67%.

**Figure 1:**
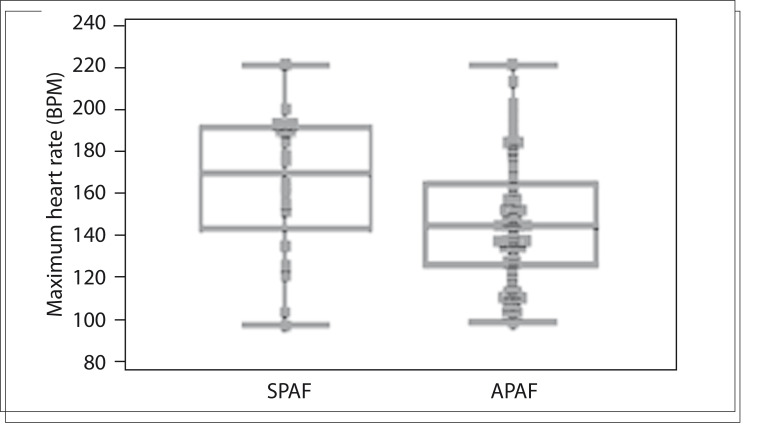
Differences between maximum heart rates, in beats per minute (BPM) in the groups with symptomatic paroxysmal atrial fibrillation (SPAF) and asymptomatic paroxysmal atrial fibrillation (APAF) (P = 0.03).

The mean age was 67 ± 13 years and 45% were female (Table 1). The patients underwent one test only. The differences between the symptomatic PAF (SPAF) and asymptomatic PAF (APAF) groups are demonstrated in Table 2. Table 3 shows the multivariate analysis on factors associated with the correlation between occurrences of PAF and symptoms. The heart rate (HR) ranged from a minimum of 45 ± 8 bpm to a mean of 74 ± 17 bpm and a maximum of 151 ± 32 bpm. Among the individuals with tests showing PAF, only 26% had symptoms. The maximum HR was higher in the group with a symptomatic correlation with arrhythmia (165 ± 34 versus 147 ± 30 bpm) (P = 0.03), as shown in Figure 1. Use of beta blockers had a protective effect against PAF symptoms (odds ratio, OR: 0.24, P = 0.031).

**Table 1:** Baseline characteristics of the study population

Characteristics	Total
	(n = 75)
Gender: male	54.70%
Age	67.3 ± 12.5
Heart rate (bpm)	
Minimum	45.8 ± 8.4
Mean	74 ± 16.8
Maximum	151.9 ± 31.9
Symptomatic paroxysmal atrial fibrillation	26%
Drugs	
Beta blockers	36.60%
Sotalol	4%
Diuretic	20%
Digoxin	2.70%
Amiodarone	18.70%
Calcium channel blockers	17.30%
Angiotensin receptor blockers	5.30%
Angiotensin-converting enzyme inhibitors	22.70%
Statin	9.30%
Oral anticoagulants	5.30%
Acetylsalicylic acid	28%
Others	30.70%

**Table 2:** Characteristics of the groups with symptomatic paroxysmal atrial fibrillation (SPAF) and asymptomatic paroxysmal atrial fibrillation (APAF)

Characteristics	SPAF (n = 20)	APAF (n = 55)	P
Gender	5.3	18.8	0.28
Age	66.5 ± 15.8	67.6 ± 11.1	0.08
Number of episodes	4.6 ± 5.5	11.6 ± 29.6	0.1
Number of drugs	3.3 ± 1.5	2.4 ± 2.0	0.16
Prevalence during wakefulness	75%	81.80%	0.52
Duration of atrial fibrillation (min)	120	122	0.81
Maximum heart rate (bpm)	164 ± 7.3	164 ± 7.3	0.03^*^

* Significance obtained from analysis of covariance (ANCOVA) with estimated means (± standard error) and adjusted according to the factors of age, gender, number of episodes, number of drugs and heart rate.

**Table 3:** Multivariate analysis on factors associated with the correlation between occurrences of paroxysmal atrial fibrillation and symptoms

Variable*	Odds ratio	P	95% confidence interval	
Increment of 1 bpm in maximum heart rate	1.022	0.015	1.004	1.040
Use of beta blocker	0.246	0.031	0.068	0.883

* Variables placed in the model backwards were: age, gender, minimum heart rate, mean heart rate, maximum heart rate, use of beta blocker, sotalol, amiodarone, digoxin, atrial fibrillation during wakefulness, duration of atrial fibrillation episode in minutes

bpm = beats per minute.

**Figure 2:**
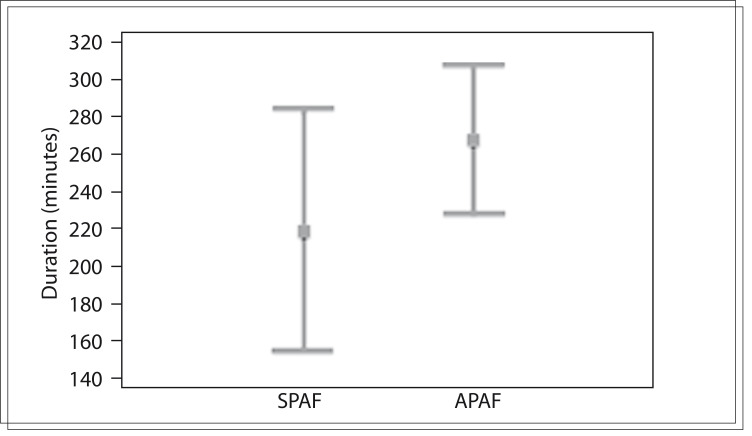
Number of episodes of paroxysmal atrial fibrillation in atrial fibrillation (SPAF) and asymptomatic paroxysmal atrial fibrillation (APAF) (P = 0.36).

The duration in minutes of the episodes of PAF was similar in the two groups (P = 0.53), as shown in Figure 2. There was no difference in the number of episodes of PAF in the two groups (P = 0.36), as shown in Figure 2.

## DISCUSSION

There is evidence showing that atrial fibrillation, which was once considered to be a benign arrhythmia, is a condition that causes significant morbidity.^2^^,^ 3 It has become a public health problem because of the aging of the population and the increasing prevalence of degenerative diseases. The subjects of the present study who showed PAF during the 24-hour Holter test were of relatively high average age and presented comorbidities resulting from chronic diseases.

Even though our study was conducted on a selected population of patients for whom a Holter test was indicated, the prevalence of PAF was similar to that reported in the literature.^8^^,^ 9 The presence of symptoms in patients with PAF is of fundamental importance because this is the criterion for curative treatment of such arrhythmia, whether by surgical means or by catheter ablation. Identification of asymptomatic PAF is also important, since the need for antiplatelet drugs or anticoagulant medication for preventing thromboembolic events has to be assessed. Symptoms were present during the Holter test in 59% of the patients who were found to have PAF. However, a correlation between the episode of arrhythmia and the reported symptom was present in only 26% of the cases.

The perception of symptoms changed after ablation inpatients were found to have symptomatic AF, as demonstrated by Hindricks et al.^10^ Increased incidence of asymptomatic AF was detected in the Holter test after the procedure. Furthermore, the results from this study showed that asymptomatic PAF may occur in symptomatic patients who present an indication of PAF ablation. Before ablation, 50% of the patients had either symptomatic or asymptomatic PAF, while 38% of the patients recognized precisely all episodes of PAF. Even with a history of symptomatic PAF,^10^ only 5% of the patients had asymptomatic PAF during electrocardiographic recordings over seven days.

Jabaudon et al.^11^ compared the detection of AF with the incidence of cerebral thromboembolic events, because of the importance of such occurrences. They analyzed arrhythmia through electrocardiographic recordings over a seven-day period (event-loop recording), in addition to basic electrocardiography and Holter tests after the occurrence of the episodes. AF was found in 2.7% of the basic electrocardiograms, 5% of the Holter tests and 5.7% of the event-loop recordings. The basic electrocardiographic and Holter results were normal^11^ in patients who were diagnosed by means of the latter method.

The Discern AF Trial study was applied to evaluate the follow-up on patients who had been referred for AF ablation, with an implantable event monitor over the three-month period before the procedure. The device was able to diagnose 69% of the 2355 apparent episodes of arrhythmia as AF or atrial flutter. During this period, the device was activated 5013 times. According to the monitor, only 47% of the patients were confirmed by the event monitor, whereas 46% of them remained relapse-free after ablation.^12^

The current drug treatment or non-pharmacological approaches may offer partial or complete relief of the symptoms. However, this may lead to the belief that AF has been completely controlled or even cured, due to the absence of symptoms or documentation of sinus rhythm through electrocardiography or occasional 24-hour Holter tests. Another important point is that the use of beta blockers drugs provides protection against occurrences of symptoms relating to PAF, probably because of their negative chronotropic property. 

Another important finding from this study was the positive symptomatic correlation in patients who presented higher HR during the 24-hour recording. This was evident even when the analysis was controlled for the use of antiarrhythmic and negative chronotropic drugs. Although the Holter test was used to check HR over a 24-hour period, it showed that the HR during episodes of AF corresponded to the maximum HR in most tests.

The limitations of this study included the selection bias inherent to the study design, the possibility that patients might present some pathological condition or symptoms indicating they should undergo a 24-hour Holter test, lack of information regarding the indication of tests and the exact use of antiarrhythmic drugs. The daily journal of symptoms filled out by patients also represented a limitation, given that the patients might omit important symptoms or overvalue them. This certainly occurs, as was proven in a study on long-term electrocardiographic monitoring over a seven-day period, among patients with atrial fibrillation.^13^

One of the main implications for clinical practice from the present study is that a symptom-based approach towards evaluating PAF is unreliable. Our study showed that symptoms are not only related to PAF per se; HR seems to be an important factor too. PAF with a slow HR may be missed during a routine clinical visit.

This study provides the notion that curative treatment has to be followed by prolonged monitoring. Future investigations should be directed towards better understanding of the factors that contribute to occurrences of symptoms in PAF. Holter monitoring is a valuable tool within this scenario.

## CONCLUSION

Detection of asymptomatic episodes of PAF in patients who underwent a 24-hour Holter test was a uncommon event. These results were in accordance with the literature and demonstrated that there was a low correlation between symptoms and episodes of PAF during Holter recording.

The prevalence of PAF was similar to that found in the literature. These events were often asymptomatic in this selected population of patients. The maximum HR during the arrhythmia episode was the only factor related to the presence of symptoms during the arrhythmia event. Use of beta blockers served as a protector against occurrences of symptoms in patients with PAF.
